# Advances and Challenges in the Hunting Instability Diagnosis of High-Speed Trains

**DOI:** 10.3390/s24175719

**Published:** 2024-09-02

**Authors:** Jiayi Liang, Jianfeng Sun, Yonghua Jiang, Weifang Pan, Weidong Jiao

**Affiliations:** 1 School of Engineering, Zhejiang Normal University, Jinhua 321004, China; 2 Xingzhi College, Zhejiang Normal University, Lanxi 321100, China

**Keywords:** high-speed trains, hunting instability, fault diagnosis, signal analysis, transfer learning

## Abstract

With the continuous increase in train running speeds and the rapid complexity of operation environments, running stability of the high-speed train is facing significant challenges. A series of abnormal vibration issues, caused by hunting instability, have emerged, including bogie instability alarm, carbody swaying, and carbody shaking, posing a significant threat to the safe and stable operation of high-speed trains. Therefore, the monitoring and diagnosis of hunting instability have become important research topics in rail transit. This review follows the development of fault diagnosis for bogie hunting instability and carbody hunting instability. It first summarizes the existing evaluation standards and innovative diagnostic methods. Due to the current limitation of hunting instability evaluation standards, which can only detect large-amplitude hunting, this paper addresses the gap in evaluation criteria for early-stage, small amplitude hunting instability diagnosis. A thorough overview of the progress made by researches in this field of research is given, emphasizing three primary facets: diagnostic signal sources, diagnostic features, and diagnostic targets. Furthermore, given that existing methods only classify faults into small and large amplitudes, which does not meet the practical need for quickly and accurately identifying fault types and severity during operation, this review introduces existing works on the detailed assessment and fault tracing of hunting instability, as well as the mechanisms underlying its occurrence, with the aim of achieving a comprehensive diagnosis of hunting instability. Finally, the limitations of current methods and the future development trends in hunting instability diagnostics are discussed and summarized. This paper provides readers with a framework for the research process of hunting instability diagnosis, offering valuable references and innovative perspectives for their future research efforts.

## 1. Introduction

High-speed trains represent a crucial component of national infrastructure and a popular mode of transportation. They offer significant advantages in terms of energy consumption, low carbon emissions, and transportation efficiency, making them an ideal solution for the urgent development of green transportation [1]. As of the end of 2023, China operated a fleet of 4133 standard trainsets, with over 260,000 wheels in service across 45,000 km of dedicated passenger lines. The highest operating speed reached 350 km/h, with high-speed trains capable of exceeding 400 km/h currently under development. Due to the continuous increase in train operating speeds and the long-term impacts of wheel-rail wear, suspension component failures and performance degradation, improper track maintenance, and changes in the operating environment have led to significant temporal and spatial variations in the wheel-rail contact relationship, suspension performances, and excitation loads on various components under service conditions. These factors pose major challenges to train running stability, resulting in a series of abnormal vibration issues caused by hunting instability (including bogie instability alarms [2,3,4], swaying [5,6,7], and shaking [8,9,10,11]), which significantly threaten the safe and stable operation of high-speed trains.

Hunting instability is a dynamic instability phenomenon that occurs when railway vehicles exceed a critical speed. It manifests as a periodic motion involving the coupling of lateral displacement and yawing of the wheelset [12], which induces harmonic vibrations in the bogie and can lead to localized cracks [13]. This vibration is transmitted upward to the carbody, resulting in lateral swaying or shaking that deteriorates ride comfort [8,14]. In severe cases, it can lead to collisions between the wheels and rails, resulting in track deformation and increased derailment risks [15,16]. During the initial design phase, high-speed trains are engineered to exhibit perfect hunting stability. However, with prolonged service, wheel-rail wear and external factors increasingly contribute to the emergence of hunting instability.

Currently, in engineering practice, hunting instability can only be temporarily controlled through wheel profile turning. In China, high-speed trains employ a fixed mileage wheel profile turning strategy, optimizing and adjusting the turning intervals based on different types of high-speed trains [17,18,19]. However, these efforts are still carried out within the traditional periodic maintenance framework. The fixed mileage wheel profile turning strategy frequently results in issues of “under-maintenance” and “over-maintenance” of the wheels. This approach can pose risks to train operation safety, diminish operational efficiency, and lead to substantial waste. To ensure the safety and reliability of high-speed trains throughout their entire life-cycle while optimizing maintenance costs, the online monitoring and diagnosis of hunting instability are essential. Consequently, it is crucial to conduct a comprehensive review of recent research, highlight advancements in hunting instability diagnosis, and explore future trends and potential solutions to emerging challenges.

This review provides a novel framework for understanding and clarifying the current state of research in the field of hunting instability fault diagnosis. By examining the developmental trajectory of this field, it identifies key gaps at various stages of research. Through the integration of insights from multiple disciplines, the review proposes the potential application of transfer learning as a new research avenue. This work aims not only to summarize existing knowledge but also to advance the field of hunting instability fault diagnosis, offering valuable guidance for future research endeavors. The specific arrangement is as follows: the research background on hunting instability diagnosis has been introduced, and the critical need for intelligent diagnostics in this field has been emphasized in Section 1. In Section 2, a comprehensive summary of existing official standards for determining hunting instability, has been provided. Given that official standards have only been able to identify and detect significant hunting instability, in Section 3, a detailed introduction to the research conducted by scholars on the detection methods and processes for early-stage small-amplitude hunting instability from three different perspectives has been provided. In Section 4, existing research on the fine-grained assessment and fault tracing of both bogie and carbody hunting instabilities has been reviewed, with an emphasis on their significance in diagnosing hunting instability. It also analyzes how the lack of clarity regarding fault mechanisms has been a primary reason for the inability to conduct detailed assessments and fault tracing. In Section 5, the mechanisms of hunting instability are explored through three aspects: the influencing factors and patterns of hunting instability, the degradation mechanisms of vehicle dynamics, and the development of common characteristics associated with hunting instability. The final Section summarizes the entire paper, identifies the shortcomings of current fault diagnosis research, proposes potential integration with transfer learning, and outlines future research directions.

## 2. Evaluation Criteria for Vehicle Hunting Instability

The stability of hunting instability is crucial for the safe and stable operation of high-speed trains. Hunting instability must be prevented during daily train operations, as it can compromise vehicle stability and, in severe cases, result in derailment. To ensure running safety and stability, it is essential to perform the online monitoring and quantitative assessment of hunting instability. This necessitates the development of standards or guidelines to define evaluation methods, metrics, and thresholds [20].

### 2.1. Evaluation Methods Based on Wheel-Rail Guiding Forces and Axle-Box Lateral Forces

Guiding forces and axle-box forces directly reflect the impact of hunting instability on vehicle operating safety and are therefore commonly utilized as signal sources for evaluating and diagnosing train hunting stability. The international standards for railway vehicle dynamics, EN 14363 [21] and UIC 518 [22], specify detailed guidelines for the collection, filtering methods, and instability thresholds of guiding forces and axle-box forces: the sum of wheel-rail guiding forces ∑Y or the axle-box lateral forces *H* should be processed through a bandpass filter with a bandwidth of $f_{0}\pm 2$ Hz, where *f*_0_ represents the hunting frequency. Subsequently, the root mean square (RMS) value is computed using a sliding window of 100 m in length and a step size not exceeding 10 m. The threshold is set at *k*(10 + *P*_0_/3)/2 kN, where the coefficient *k* depends on the type of force, vehicle type, and vehicle load, and *P*_0_ represents the static axle load. However, in practical applications, the technical costs and complexities associated with acquiring guiding forces or axle-box forces are high, which makes it challenging to implement these methods on a broad scale in routine operations.

### 2.2. Evaluation Methods Based on Bogie Frame Acceleration and Carbody Acceleration

The lateral acceleration of the bogie frame directly reflects the dynamic behavior of the bogie. Additionally, the cost of signal-acquisition devices is relatively low, and their installation is straightforward. Consequently, most existing evaluation standards rely on the lateral acceleration signals of the bogie frame to diagnose the occurrence of hunting instability. Various methods can be used to evaluate hunting instability through these acceleration signals, with the root mean square (RMS) method and the peak value method currently employed in the standards.

The root mean square (RMS) method employs the RMS value as the evaluation metric. Standards EN 14363 [21] and UIC 518 [22] provide specifications for evaluating acceleration using the root mean square (RMS) method as follows: acceleration signals must be filtered through a bandpass filter with a bandwidth of $f_{0}\pm 2$ Hz. The RMS value is then computed using a sliding window of 100 m in length with a step size not exceeding 10 m. The threshold is set at (12 − *M_b_*/5)/2 m/s^2^, where *M_b_* denotes the mass of the bogie in tons. Standard 49CFR 213 [23] mandates that acceleration signals undergo low-pass filtering at 10 Hz and a detrending process. The RMS value is subsequently calculated using a sliding window of 2 s in duration. The amplitude must not exceed 0.3 g over a continuous duration of 2 s.

The peak value method for acceleration assesses instability by counting the number of instances where lateral acceleration peak values continuously exceed a specified limit. Standard UIC 515-1 [24] specifies that, according to the peak value method for acceleration, the peak values, after being processed through a 10 Hz low-pass filter, must not exceed 8~10 m/s^2^ continuously over six instances. Standard TSI RST HS 232 [25] specifies that, after being processed through a bandpass filter with a frequency range of 3 to 9 Hz, the peak values of acceleration must not exceed 0.8 g continuously across 10 instances. The Chinese standard GB/T 5599-2019 [26] specifies that, after being processed through a bandpass filter with a frequency range of 0.5 to 10 Hz, the peak values of acceleration must not exceed 8 m/s^2^ continuously across six instances.

**Figure 1:**
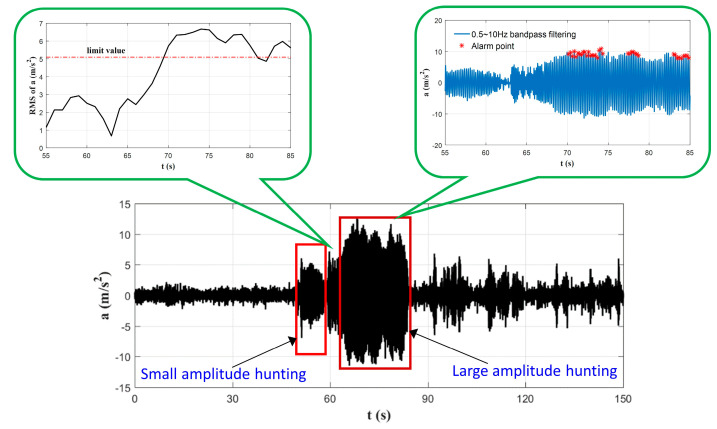
Time-domain signals and the assessment results for the two methods based on the bogie frame lateral acceleration [27].

However, these standards (as shown in Table 1) are primarily intended for vehicle certification testing and do not consider the impact of hunting instability on vehicle dynamic performance. As shown in Figure 1, the existing standard employs a fixed value for evaluation, which can only identify significant hunting instability and fails to detect small-amplitude hunting instability. This limitation prevents accurate fault identification, making it unsuitable for online monitoring and diagnosis of the vehicle’s operational conditions.

## 3. Early Detection of Hunting Instability

Currently, there are no official standards for the evaluation of small-amplitude hunting instability. An analysis of the monitoring data demonstrates that, prior to the onset of significant hunting instability, high-speed trains typically manifest small-amplitude hunting instability. This motion is characterized by a well-defined periodic pattern, with amplitudes that exceed normal levels but fall below the threshold for severe instability. In some papers [27,28,29,30], it is defined as follows: when the wheelset exhibits small-amplitude displacements, the amplitude of lateral-acceleration oscillations does not meet the thresholds specified by evaluation standards, yet distinct hunting oscillations are observed. Early-stage small-amplitude hunting motion can negatively impact the comfort of passengers and drivers, accelerate wheel-rail wear, and, over extended periods of train operation, potentially escalate into significant hunting instability, thereby compromising the safety of train operations. Therefore, the timely detection of early-stage hunting motion is of critical importance. To address this issue, numerous researchers have undertaken comprehensive studies on the diagnosis of early-stage hunting instability using signal-analysis techniques and artificial intelligence methods. The subsequent summary will be presented from three perspectives: (1) diagnostic signal sources; (2) diagnostic features; and (3) diagnostic targets.

### 3.1. Diagnostic Signal Sources

Signal acquisition plays a crucial role in fault diagnosis systems, as the quality of the signal directly determines the accuracy and reliability of the diagnostic results. In the fault diagnosis process, accurate and complete signals enable the diagnostic system to precisely extract root cause information, thereby enhancing diagnostic accuracy and providing robust support for the reliable operation and effective maintenance of the system. Therefore, when designing a fault diagnosis system, it is essential to meticulously consider both the quantity and placement of sensors to ensure comprehensive signal acquisition and maintain the completeness of the information.

**Figure 2:**
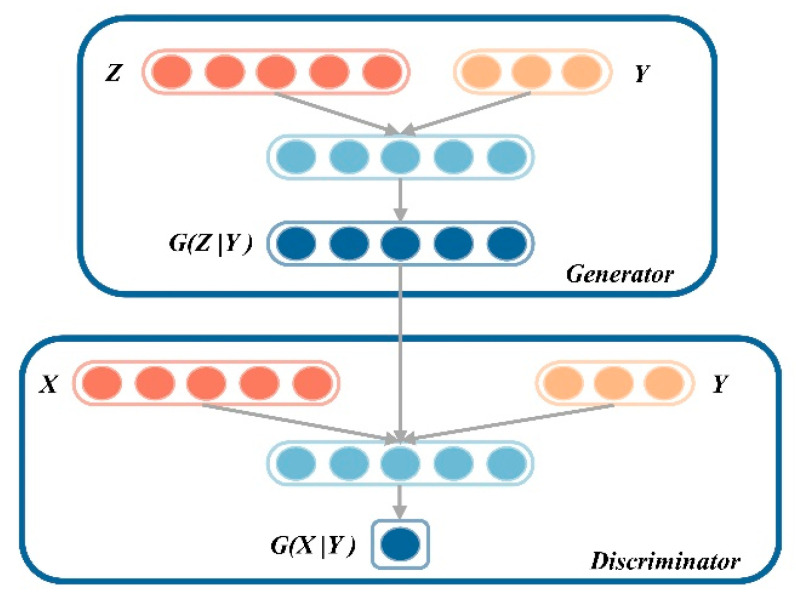
Condition GAN.

The lateral acceleration measured at the ends of the bogie frame can simultaneously reflect both the rocking and lateral displacement modes of the bogie. Most existing research relies on this singular signal source (the lateral acceleration of the bogie frame) to diagnose the occurrence of hunting instability. Bruni et al. [31] defined the stability region of hunting motion based on online measured acceleration by employing the Random Decrement Technique (RDT) and Prony’s method (complex exponential model). This approach avoids the traditional numerical modeling process and directly estimates the hunting frequency from the autocorrelation function, enabling the effective identification of early hunting instability. Li et al. [32] developed a novel instability detection system for EMUs based on bogie vibration signals and adjusted the alarm threshold for small-amplitude hunting to $5\;m\cdot s^{-2}$. In [33], Wang et al. focused on bogie frame acceleration signals and proposed a research method based on 1D CNN and LSTM, which effectively identifies small-amplitude instability. Ning et al. [34] collected lateral acceleration signals from high-speed train bogies for their study. To address the issue of data imbalance in the measurements, they proposed a prediction method based on 1D-CNN and CGAN (the structure of the CGAN network is illustrated in the Figure 2). The core of this method lies in utilizing the CGAN model to augment the original data sample space, effectively addressing the significant challenge of sample imbalance in diagnostic tasks. With a prediction accuracy of up to 97.5%, this method demonstrates substantial superiority over several comparative approaches and is highly effective in providing early warnings of hunting instability. However, this model is limited to fitting the original data rather than generating new, authentic data. As a result, the sample space after data augmentation remains quite limited in diversity.

**Figure 3:**
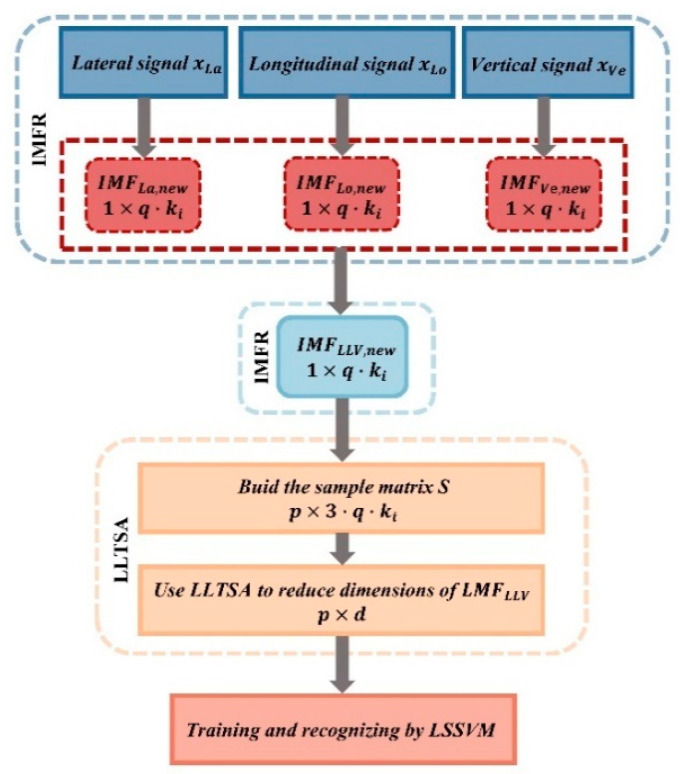
Technical route of combining the bogie frame’s lateral–longitudinal–vertical data fusion and IMFR-LLTSA [29].

The aforementioned studies predominantly employ single-signal approaches for fault diagnosis. However, hunting instability typically entails a complex nonlinear relationship wherein multiple factors contribute to diverse outcomes. Relying solely on data from a single measurement point is insufficient for effective fault tracing and diagnosis. Consequently, numerous researchers have investigated multi-source information fusion techniques within the domain of hunting motion diagnostics. Given that a single sensor cannot fully capture the operational conditions of the bogie, Liu et al. [35] employed a network system incorporating multiple acceleration sensors to develop a method for identifying high-speed train lateral instability fault states using D-S evidence theory. Compared to any single sensor diagnosis, his approach exhibits superior performance in distinguishing normal conditions, small-amplitude hunting motion, and significant hunting fault states. It represents a pioneering application of multi-sensor fusion technology in high-speed trains. Building upon Liu’s research, Ning [36] introduced an advanced multi-sensor fusion framework utilizing an enhanced D-S theory. This framework integrates empirical mode decomposition (EMD) and sample entropy methods to extract non-stationary features from the data provided by each sensor. It constructs a posterior probability support vector machine (PPSVM) model to derive basic probability assignments, which are then refined using the improved D-S theory for weighting. This method yielded results with exceptionally high confidence levels. The multi-sensor data fusion system significantly enhances system reliability and robustness, extends the observational range across both temporal and spatial dimensions, and improves the credibility of the data, as well as the resolution of the system. However, Ning’s method relies exclusively on lateral acceleration signals to evaluate the fluctuation state, while it does not fully integrate longitudinal and vertical vibration signals. Therefore, Ye et al. [29] proposed a method that combines lateral-longitudinal-vertical data fusion from the bogie frame with IMFR-LLTSA. This approach utilizes EEMD to decompose vibration signals from the three directions of the bogie frame, reconstructing them into a new vector, IMFLLV. The LLTSA is then employed to obtain an adaptive low-dimensional feature matrix, effectively utilizing the nonlinear dimensionality reduction capabilities of manifold learning to extract inherent structures from high-dimensional datasets, thereby mitigating the challenges associated with feature selection, and the technical scheme is illustrated in the Figure 3. This method was applied to CRH380A high-speed trains operating on the Shanghai-Hangzhou line. Experimental results demonstrated that this approach significantly outperforms other methods relying solely on lateral acceleration. Sun et al. [27] proposed a simple, efficient, and highly cost-effective algorithm that relies solely on basic signal analysis techniques. The algorithm collects nine vibration signals from different parts and directions of the train’s structure and body and pairs them to form correlation indices. By analyzing the variation patterns of these indices during the fault evolution process, the algorithm identifies the most indicative indices for detecting hunting instability. It not only detects small-amplitude instabilities but also distinguishes between the two different states of small-amplitude instability: small-amplitude divergence and small-amplitude convergence. To investigate the hunting motion in bogies of subway trains operating at relatively low speeds, Zheng et al. [37] conducted field tests to measure the vibration accelerations of the axle-box, bogie frame, and car body floor. They also measured the relative displacements between the frame and the wheelset and between the brake caliper and the wheelset, to evaluate the vehicle’s vibration characteristics. In [38], Kulkarni introduced an indicator, *CHI*, which integrates phase and amplitude information from both lateral and longitudinal axle-box accelerations to detect coupling modes in the lateral and yaw directions. Under unstable wheel-rail contact conditions, the phase angle difference between longitudinal and lateral accelerations remains unaffected, allowing this method to effectively identify hunting states in practice. However, since the phase angle difference between these two accelerations varies depending on the vehicle, applying the *CHI* index without a specific vehicle model becomes a highly challenging task. The *CHI*, obtained by multiplying *FHI* and *PDHI*, can independently assess the dynamic instability of each wheelset.

(1) CHI=FHI∗PDHI

The Frequency Hunting Index (*FHI*) quantifies the dominance of frequency *P_f_* in the dynamic response of the vehicle’s axle-box in the longitudinal (*X*) and lateral (*Y*) directions. The Phase Difference Hunting Index (*PDHI*) measures the proximity of the phase difference θ between the *X* and *Y* direction accelerations at frequency *P_f_* to a characteristic phase angle $\theta_{\mathrm{Hunting}}$. These indices are calculated using the following formulas.

(2) $$FHI=avg\left(\frac{A_{X}\left(P_{f}\right)}{\max\left(A_{X}\right)},\frac{A_{Y}\left(P_{f}\right)}{\max\left(A_{Y}\right)}\right)$$

(3) $$PHDI=1+S\left(\alpha \left(\theta -\left(\theta_{Hunting}-\theta_{o}\right)\right)\right)-S\left(\alpha \left(\theta -\left(\theta_{Hunting}+\theta_{o}\right)\right)\right)$$

where *A_X_* and *A_Y_* denote the spectral amplitudes of acceleration in the *X* and *Y* directions, respectively, and *S* represents the Sigmoid function. The gradient and the inflection point’s location on the *θ* axis in the vicinity of *θ*_Hunting_ are regulated by the parameters *α* and $\theta_{o}$ of the sigmoid function.

By incorporating multiple signal sources, the method enhances the robustness of hunting instability diagnostics and enables the accurate identification of various states of hunting instability.

Single-signal and multi-source signal systems each present distinct advantages and limitations. Single-signal systems are relatively straightforward in terms of hardware installation and data processing, offering benefits, such as lower cost and better real-time performance. However, single-signal systems offer limited information and are more vulnerable to noise, which can constrain the diagnostic accuracy and make it challenging to address complex fault modes. In contrast, multi-source signal systems, which use multiple sensors to collect multidimensional data, can comprehensively reflect the system’s operating conditions, thereby enhancing diagnostic accuracy and reliability. Nevertheless, multi-source signal systems are relatively complex, involving higher hardware and maintenance costs, as well as greater computational demands for data processing, which may impact real-time performance. Therefore, in the practical diagnostics of hunting instability, it is essential to weigh the advantages and disadvantages of single-source versus multi-source signal systems according to the specific diagnostic needs and select the most suitable approach to achieve optimal fault diagnosis outcomes.

### 3.2. Diagnostic Features

In the diagnostic process, fault features serve as the bridge between data and state recognition. Their importance lies not only in distinguishing and locating faults and predicting potential issues but also in enhancing diagnostic accuracy and precision. Appropriate features exhibit higher sensitivity to the health status of equipment, providing a clear signal background and reducing noise and interference. This facilitates the easier detection and analysis of faults, thereby supporting the maintenance and management of equipment and systems. Therefore, in the study of early-stage hunting instability diagnostics, researchers have developed a variety of discriminative methods based on different types of features. This section will review the application of time-domain features, frequency-domain features, nonlinear features, and data-driven features in the context of early-stage hunting instability.

Time-domain methods assess small-amplitude hunting movements by analyzing changes in vibration signals over time. To detect bogie hunting instability, Song et al. [39] proposed an indirect monitoring method for wheelset lateral displacement based on axle-box vibration signals. They used singular value decomposition of the Hankel matrix to calculate the amplitude of lateral movement corresponding to the hunting instability, thereby assessing the degree of instability. Yao et al. [40] comprehensively considered the effects of forced vibrations from track irregularities and self-excited vibrations due to bogie hunting instability, proposing a new lateral stability assessment method based on the root mean square (RMS) of the lateral acceleration of the frame, which introduces two key parameters, $R_{MS}^{F}$ and $R_{MS}^{H}$. When these parameters exceed the specified thresholds, the system is identified as being in a hunting state. The algorithm is straightforward and well-suited for practical engineering applications.

(4) $$\left\{ \begin{array}{l} R_{MS}^{F}\leq \frac{1}{2}k{\left(s{\ddot{y}}^{+}\right)}_{\lim}\\ R_{MS}^{H}>\frac{1}{2}k{\left(s{\ddot{y}}^{+}\right)}_{\lim} \end{array}\right.$$

where $R_{MS}^{F}$ represents the RMS value within the frequency range of 2 to 3.5 Hz, calculated over a distance of 100 m with a step length of 10 m. $R_{MS}^{H}$ represents the RMS value within the frequency range of 3.5 to 5 Hz, and *k* is the reduction coefficient for $R_{MS}$.

Frequency-domain methods diagnose hunting instability by performing spectral analysis of the signal and extracting the energy distribution of various frequency components. Guo et al. [41] integrated the spectral frequency spread and autocorrelation coefficient with a decision tree model to detect small-amplitude hunting instability, achieving an accuracy of 99.94%. Wang et al. [42] introduced an energy-based method for diagnosing hunting instability using the Hilbert-Huang Transform (HHT). This approach facilitates both the qualitative assessment of hunting motion in high-speed trains—through an analysis of the dominant frequency magnitude, spectral concentration, and frequency values—and provides a quantitative measure of the severity of hunting instability.

The nonlinear feature method determines hunting instability by characterizing the complex dynamic behavior of the signal. Zeng et al. [43] introduced a novel indicator, PIPT (Periodic Indicator of Phase Trajectories), which describes the periodicity of state variables in nonlinear dynamical systems, starting from the Lyapunov exponent. Ning et al. [28] proposed a method combining Multi-Scale Permutation Entropy (MPE) and Local Tangent Space Alignment (LTSA) to extract nonlinear features. This method was applied at high speeds of 320–350 km/h to differentiate between various states of complex signals. Compared to features based on the energy frequency distribution of bogie vibrations, the proposed method more effectively identifies bifurcation evolution in small-amplitude hunting signals. Ran [44] utilized the EEMD-SVD-LTSA method for the feature extraction of small-amplitude hunting instability in high-speed trains. The proposed method initially decomposes the signal using EEMD to obtain IMF components. Compared to traditional EMD, EEMD adds white noise to the original signal, making the signal more uniform and effectively suppressing the mode mixing caused by intermittent high-frequency components. The decomposition process is detailed as follows:

(1) Assume original signal is *m*(*t*), and add a set of Gaussian white noise *v*(*t*) to it.
  (5) $$m^{\prime }\left(t\right)=m\left(t\right)+v\left(t\right)$$
(2) Perform traditional EMD decomposition on the signal to obtain the IMF components *b_i_*, where *q* is the residual term and *n* is the number of decomposed IMF components.
  (6) $$m^{\prime }\left(t\right)=\sum_{i}^{n}b_{i}+q$$
(3) Each time, add different white noise sequences *v_i_*(*t*), with the same amplitude, and repeat the previous two steps.
  (7) $${m^{\prime }}_{j}\left(t\right)=\sum_{i=1}^{n}b_{ij}+q_{j}$$
(4) By repeating the EMD process multiple times and averaging the results, the impact of white noise can be eliminated. The IMF components corresponding to the original signal can then be expressed as follows:
  (8) $$c_{i}\left(t\right)=\frac{1}{N}\sum_{j=1}^{N}c_{ij}\left(t\right)$$
  where *N* represents the number of times white noise is added.

**Figure 4:**
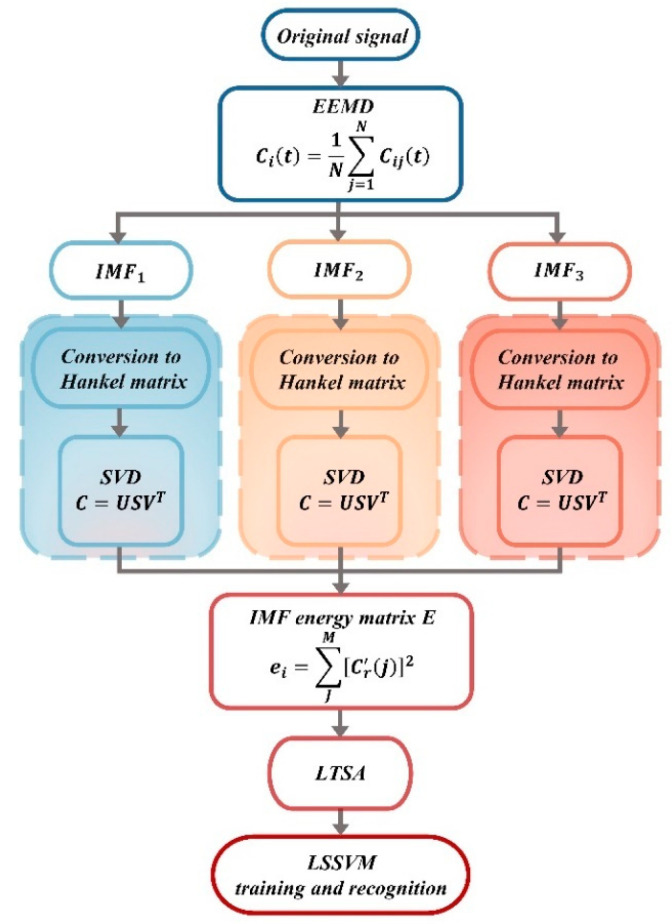
Framework process for small-amplitude hunting feature extraction.

After obtaining the IMF components, perform SVD denoising and LTSA dimensionality reduction to extract features. The framework for feature extraction is illustrated in Figure 4. Experimental results demonstrate that features extracted using this framework can successfully identify whether the evolution trend is small-amplitude divergence or small-amplitude convergence and accurately predict the operational status of the train. Cui et al. [45] employed a feature extraction methodology based on Empirical Mode Decomposition (EMD) in conjunction with manifold learning and compared it to a feature extraction approach using wavelet transform. Their results indicated that features extracted via the EMD-ISOMAP technique exhibited superior performance in distinguishing small-amplitude hunting instability compared to the time-frequency domain features derived from wavelet transform.

**Figure 5:**
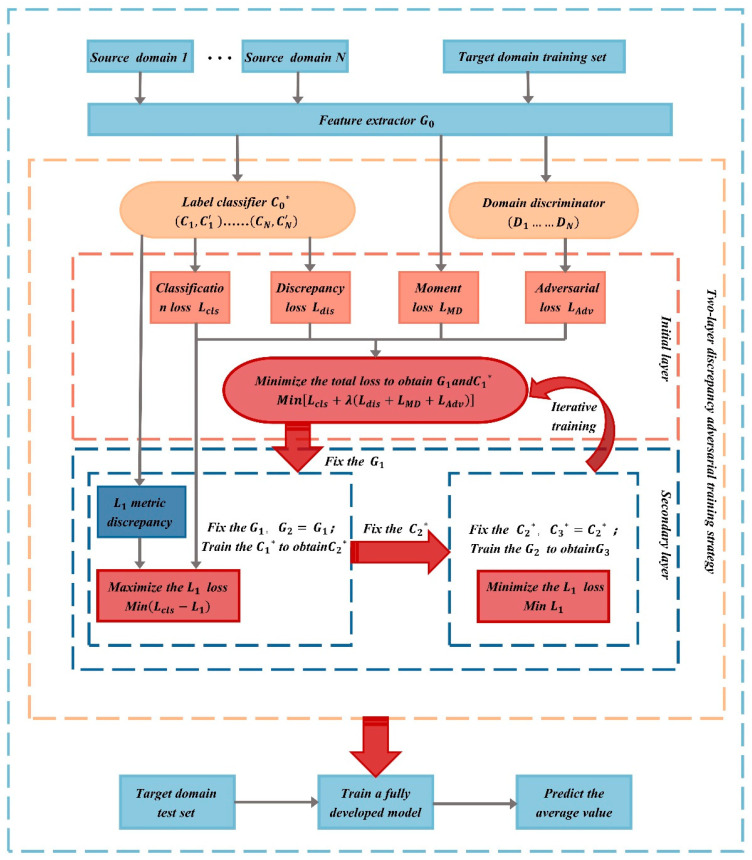
Flowchart of small-amplitude hunting state recognition based on multi-source two-layer discrepancy adversarial network.

With the rapid advancement of artificial intelligence technology, deep learning techniques for feature extraction have become increasingly prevalent across various fields. The extracted features are not limited to traditional time-domain and frequency-domain characteristics but are adaptively learned from data or images through data-driven approaches. Consequently, many researchers have increasingly applied these techniques to the recognition of high-speed train operating conditions. Liu et al. [46] proposed a method for identifying small-amplitude hunting instability in high-speed trains based on a multi-source two-layer discrepancy adversarial approach. Building on initial layer adversarial training, their method incorporates secondary layer adversarial training to better learn distinguishable features and enhance the accuracy of diagnostic tasks. A schematic diagram of their optimized feature extraction method is shown in the Figure 5. Zhao et al. [47] applied three different deep learning models to extract features from simulation data for state recognition, achieving excellent results with all methods.

Most features used to identify hunting oscillations in high-speed trains are derived from processing acceleration signals. The acceleration of the bogie frame, which results from the relative motion between the wheel and the rail, is influenced by factors, such as wheel-rail profiles, structural elasticity, and suspension parameters [48,49,50]. As such, it may not provide an intuitive reflection of the train’s safety status. Evaluating the stability of hunting oscillations from the perspective of wheel-rail dynamic interactions is more reliable. As computer vision (CV) technology came into the limelight, machine vision-based wheel–rail interaction detection methods were discovered and applied to the field of hunting state recognition. Shi et al. [51] proposed a virtual point detection method based on deep learning (DL) to detect rail wheel running dynamics (RWRD) and developed a system for measuring lateral track irregularities. Traditional image processing methods, such as edge detection and template matching, tend to suffer from performance degradation in dynamically complex backgrounds and are heavily dependent on parameter settings. To overcome these limitations, the system’s algorithm avoids traditional methods in favor of using YOLOv3-tiny to capture regions of interest (ROIs) in the input images. These ROIs are then fed into a DL model called LightPointNet, which outputs feature maps corresponding to the virtual points on the ROI. Although this method has not yet been applied to the detection of hunting motion in railway vehicles, it proves the feasibility of using DL-based virtual point detection methods to identify hunting stability. Therefore, building on Shi’s study, Ye et al. [52] applied the virtual point detection method to extract features from images for predicting and identifying the rail wheel running dynamics (RWRD) and hunting frequency. The principle of detecting hunting motion with this method involves the following: first, defining multiple virtual points on each frame extracted from the wheel-rail contact video; then, automatically detecting the coordinates of these defined virtual points using a DL model, denoting the points as *P*_1_(*y*_1_, *z*_1_), *P*_2_(*y*_2_, *z*_2_), and *P*_3_(*y*_3_, *z*_3_); and finally, calculating the relative displacement (*S*_1,*k*_ and *S*_2,*k*_).

(9) $$\left\{ \begin{array}{l} S_{1,k}=\sqrt{{\left(z_{2,k}-z_{1,k}\right)}^{2}+{\left(y_{2,k}-y_{1,k}\right)}^{2}}\\ S_{2,k}=\sqrt{{\left(z_{3,k}-z_{2,k}\right)}^{2}+{\left(y_{3,k}-y_{2,k}\right)}^{2}} \end{array}\right.$$

**Figure 6:**
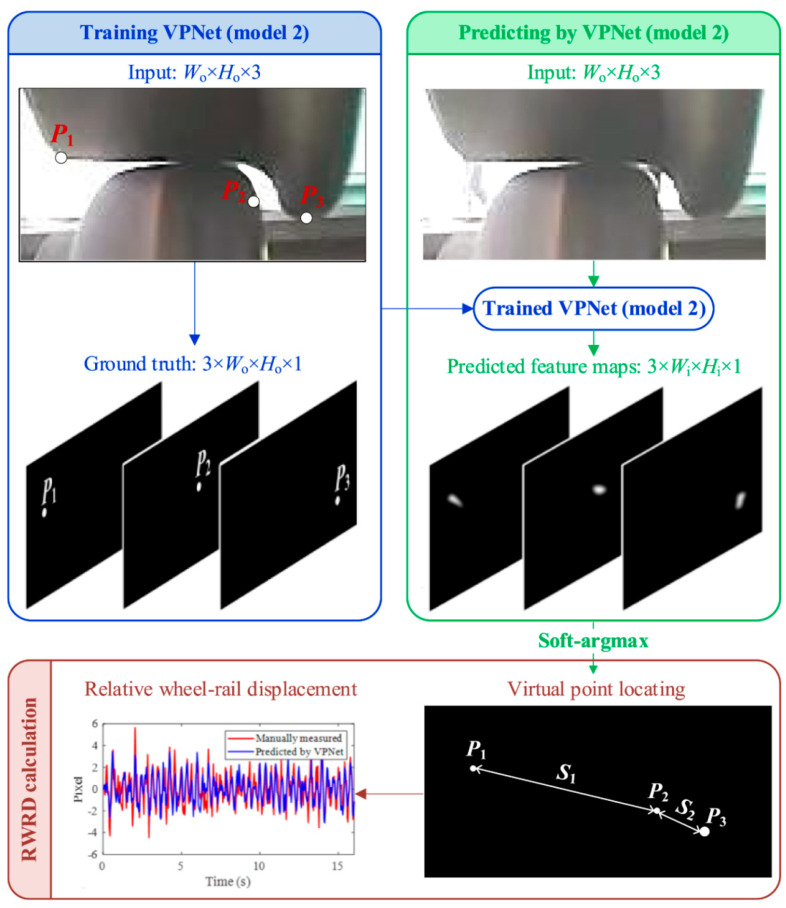
Approach to track virtual points on the wheel and rail [52].

After preprocessing the composed vectors, the determination of hunting motion is achieved by analyzing the time-domain curves or frequency distributions of these vectors. The process for assessing hunting stability using this method is illustrated in the Figure 6.

In diagnosing hunting instability, features derived from physical models and numerical analyses are not always fully applicable, as the degradation mechanisms in high-speed train systems are highly complex and cannot be accurately described by first principles. Hybrid approaches that integrate data-driven methods with model-based techniques offer a promising solution, partially overcoming the limitations of these traditional methods. Nevertheless, the effectiveness of data-driven approaches is still constrained by the challenge of obtaining a sufficiently large and representative dataset to cover all relevant operational states.

### 3.3. Diagnostic Targets

Railway vehicles are complex systems for which the design and maintenance require the consideration of multiple factors. Research from different perspectives effectively bridges theoretical studies with practical applications, enabling engineers to develop precise optimization designs and control strategies that enhance overall system stability and safety. To efficiently detect and control hunting instability, researchers have conducted extensive studies focusing on various research subjects, particularly the bogie and the carbody.

The bogie is the core component of railway vehicles, and detecting hunting motion based on the bogie has consistently been a significant topic in the study of railway vehicle dynamics. Ning et al. [53], addressing early bogie hunting instability, considered the non-stationarity of the collected bogie lateral acceleration data. They then proposed the NKJADE method, which performs feature fusion on multi-sensor lateral acceleration data, enabling the identification of early small-amplitude hunting in vehicles. In [54], based on a linearized bogie model, Guan investigated the vibrational behavior of rigid and flexible bogie hunting movements using root locus curves obtained through analytical formulas or numerical calculations. Zhao et al. [55] analyzed the simulated bogie lateral acceleration based on SIMPACK, using periodic differences in the signal and autocorrelation coefficients to identify small-amplitude hunting movements.

Unlike bogie hunting motion, carbody hunting exhibits lower frequencies, with instability predominantly occurring within 2 Hz. This frequency range significantly impacts passenger comfort [56,57], leading many researchers to concentrate on detecting hunting within the carbody. Xia et al. [58] developed a method for detecting carbody hunting by applying wavelet packet transform to raw signals. This approach extracts localized features that capture both non-stationary and short-duration signals in the time and frequency domains, thereby achieving stable and accurate predictions. Sun et al. [59] proposed a method for identifying carbody hunting instability by counting the distribution of peak values in the carbody lateral acceleration. The effectiveness of this method was validated using field measurement data.

The aforementioned studies focus on detecting either bogie hunting or carbody hunting individually. To fully leverage the variation in correlation coefficients between different vibration signals with respect to hunting instability, Sun et al. [27,60] proposed a hunting detection method based on cross-correlation technology. This method effectively selects the most representative correlation indicators of hunting instability. Given two signals *x(t)* and *y(t)*, their cross-correlation coefficient is defined as follows:

(10) $$\delta_{xy}\left(\tau \right)=\frac{R_{xy}\left(\tau \right)-\mu_{x}\mu_{y}}{\sigma_{x}\sigma_{y}}$$

where *μ_x_* and *μ_y_* are the means of the signals, *σ_x_* and *σ_y_* are the standard deviations, and $R_{xy}\left(\tau \right)$ is the cross-correlation function of the two signals, defined as follows:

(11) $$R_{xy}\left(\tau \right)=\frac{1}{N}\left(\sum_{n=1}^{N-\left|\tau \right|}x_{n}y_{n}+\tau \right),\tau =0,\pm 1,\pm 2,\ldots$$

where *N* is the number of samples considered for the signal couple.

**Figure 7:**
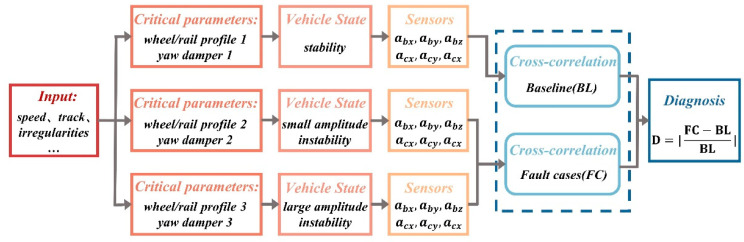
Flowchart of the proposed hunting instability detection methodology [27].

This method not only effectively identifies bogie instability but also accurately detects car body hunting, achieving the simultaneous detection of both phenomena. The diagnostic process of this method is illustrated in Figure 7.

With the rapid advancement of testing technologies and artificial intelligence, research on hunting instability diagnostics is flourishing and still in the ascendant. However, significant gaps remain. The aforementioned diagnostic methods primarily focus on determining whether hunting instability occurs but do not assess the specific degree of instability or trace the root causes. This limitation hinders their practical application in railway operations maintenance and management, failing to provide the necessary support for effective decision-making.

## 4. Evaluation and Fault Tracing of Hunting Instability

Hunting instability diagnosis encompasses three interrelated aspects: detecting the occurrence of instability, assessing the severity of instability, and tracing the contributing factors. Each of these components is crucial and must be addressed in a sequential manner. Fine-grained evaluation provides accurate and comprehensive data, enhancing the accuracy and reliability of the diagnosis. Fault localization conducts a detailed exploration of the root causes of issues, addressing fundamental problems to prevent recurrence. These two aspects complement each other, together improving system stability, reliability, and maintenance efficiency. By focusing on and strengthening both areas, the overall level of fault diagnosis and maintenance management can be significantly improved.

### 4.1. Fine-Grained Evaluation

The fine-grained evaluation of hunting instability refers to the process of conducting a comprehensive and detailed assessment of the equipment’s condition and performance through high-precision measurements, data analysis, and an in-depth understanding of the system during fault diagnosis. This evaluation is crucial for understanding the vehicle status and guiding operational decisions, making it imperative to establish a tiered assessment system for hunting instability.

**Figure 8:**
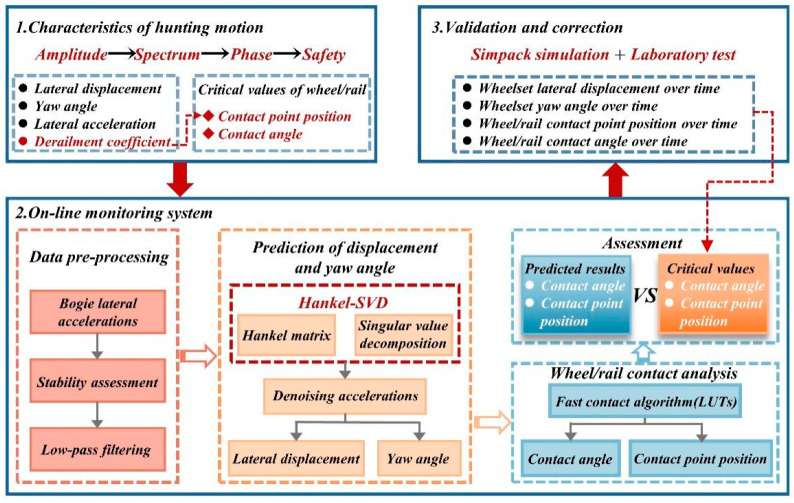
The overall structure of the research work [62].

Assessing the operational safety of high-speed trains is the primary task in conducting hunting instability diagnostics. In the absence of wheel-rail force data, some researchers have attempted to evaluate hunting instability based on the relative lateral displacement between the wheel and rail. Examples include trackside monitoring systems based on displacement sensors [61] and onboard monitoring systems utilizing computer vision [52]. In addition to directly measuring the relative displacement between the wheel and rail, Sun et al. [62] developed a method for evaluating hunting instability in high-speed trains based on lateral acceleration from the bogie. Utilizing Hankel-SVD technology, they predicted the lateral displacement and yaw angle of the wheelset and, combined with wheel-rail profile data, forecasted the wheel-rail contact relationship; the technical approach is illustrated in the Figure 8. This method, which employs only two sensors to predict the wheel-rail contact for the front wheelset of the leading bogie, achieves a balance between monitoring system accuracy and cost-effectiveness. However, this diagnostic approach is constrained by technical limitations, as the testing system is susceptible to environmental influences, such as lighting conditions, dust, rain, and track irregularities, which can affect the accuracy and resolution of displacement measurements.

**Figure 9:**
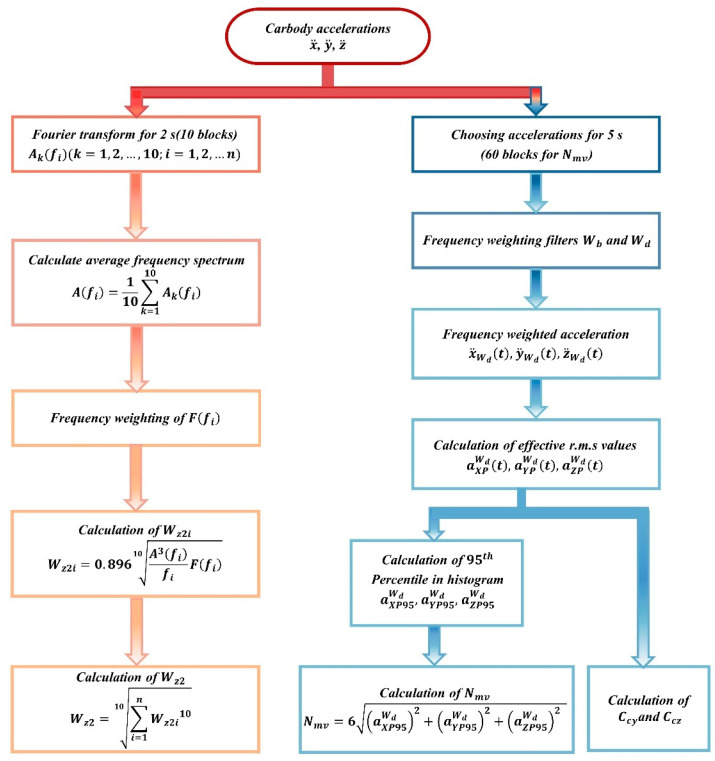
Flow chart of the ride comfort evaluation procedure of railway vehicles [57].

Additionally, focusing on passenger comfort as a baseline, Sun [57] proposed a novel evaluation method for bogie hunting instability based on peak value techniques. This method first compares the Sperling index, average comfort index, and continuous comfort index of the vehicle body acceleration during a primary hunting instability event. The analysis revealed that the Sperling index has limitations: it deviates from both the dominant frequency of carbody hunting and the sensitivity frequency of the human body in its frequency-weighted distribution, which can lead to an underestimation of the impact of carbody hunting on passenger comfort. Consequently, the continuous comfort index was concluded to be a better choice for evaluating passenger comfort under conditions of dynamic instability. Based on this method, a relationship model between *C_Cy_*, acceleration amplitude, and hunting frequency was developed to assess primary hunting events; the evaluation process flowchart is shown in the Figure 9.

### 4.2. Fault Localization

Fault localization is a systematic process designed to identify the root cause and pathways leading to system failures. It goes beyond examining surface symptoms to explore the underlying factors contributing to these symptoms, thereby enabling the implementation of effective measures to prevent recurrence. This approach is advantageous in mobilizing maintenance resources.

**Figure 10:**
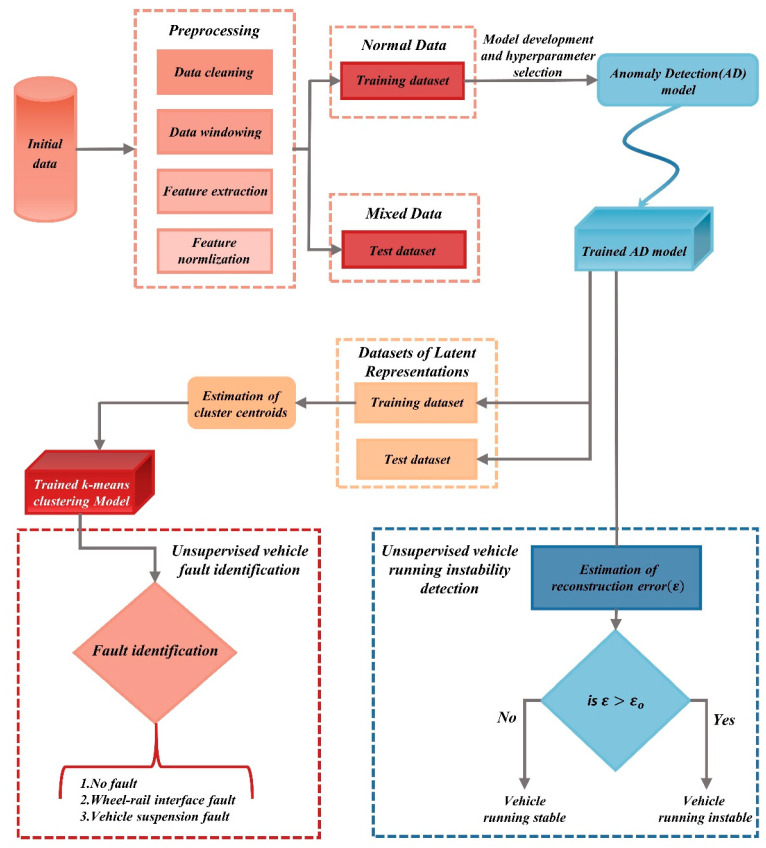
The framework of the proposed IVRIDA algorithm [64].

Bruni et al. [63] simulated various wheel-rail matching conditions and yaw damper performances to generate instability signals under different fault scenarios. They employed stochastic reduction techniques alongside the Prony method to extract fault features and then used Artificial Neural Networks (ANNs) and k-Nearest Neighbors (k-NN) methods for fault detection and isolation. Kulkarni et al. [64] introduced an unsupervised instability detection framework (iVRIDA) for identifying instability phenomena during train operations, as depicted in Figure 10. This framework employs the k-means algorithm to perform cluster analysis on the fault factors causing instability, aiming to achieve fault localization. However, the fault factors considered in the studies referenced are not comprehensive, and the effectiveness of fault localization is not ideal.

In summary, current research on hunting instability diagnosis, focuses primarily on instability detection, mainly addressing bogie hunting. There is relatively little research on fine-grained evaluation and fault localization. The reason for this is that existing studies often focus on signal-based diagnostics, which are detached from the underlying mechanisms and characteristics of the faults, making it challenging to establish a comprehensive fault evaluation system and localization mechanism.

## 5. Mechanisms of Hunting Instability

**Figure 11:**
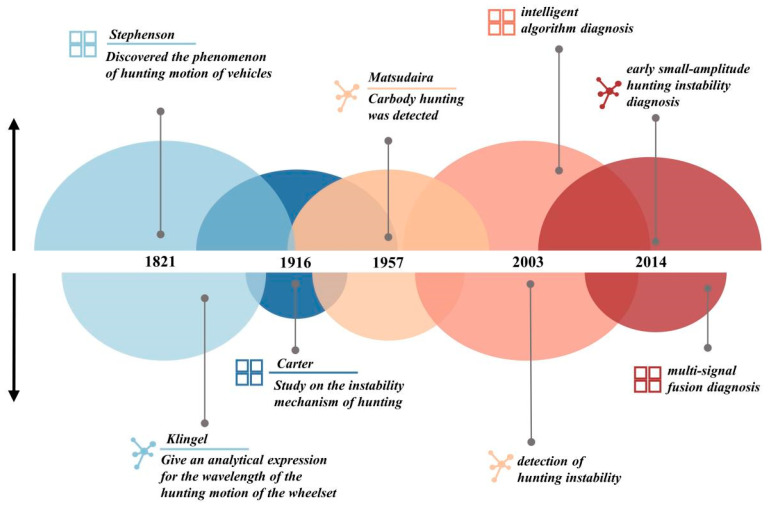
Research history of hunting stability.

Investigating the underlying mechanisms of hunting instability is crucial for accurately identifying its state. Understanding the fault mechanisms and characterization methods not only aids in uncovering the intrinsic features of the fault and establishing reasonable fault models [65] but also guides the development of effective control and mitigation measures. The hunting instability in rail vehicles has been studied extensively over the course of nearly two centuries. The historical research progress of hunting stability is illustrated in the Figure 11. In 1821, Stephenson [66] firstly observed the phenomenon of hunting motion. Later, in 1883, Klingel [67] provided the first analytical expression for the wavelength of hunting motion in wheelsets, advancing the understanding of this oscillatory behavior. In 1916, Carter [68] identified that the root cause of hunting motion lies in the creep phenomena between the tapered treads and the rail. This discovery initiated the exploration of the causes and influencing factors of hunting instability. Further advancements were made in 1957 when Matsudaira [69] discovered two distinct types of hunting motions, which he termed primary and secondary hunting; primary hunting occurs at lower speeds and is associated with the lateral motion of the vehicle body. By 2003, international standards, such as EN14363 [21], UIC518 [22], and TSI L84 [25], were established, using the filtered lateral acceleration signal of the bogie frame to determine the presence of bogie hunting instability based on whether the data exceeded a certain threshold. Recently, in 2017, many researchers [70,71,72] have been dedicated to developing methods to evaluate the level of vehicle body swaying caused by carbody hunting instability. The study of the mechanism of hunting stability remains a crucial research topic in railway vehicle dynamics. The diagnostic methods for hunting instability and the investigation of its underlying mechanisms are complementary to each other. Mechanistic research aimed at diagnosing hunting instability primarily includes the following three aspects: (1) factors influencing hunting stability and their patterns, (2) the dynamic degradation mechanisms in the case of hunting instability, and (3) the common characteristics of hunting instability.

### 5.1. Influencing Factors in Regard to Hunting Stability

Clarifying the factors that influence hunting stability and understanding their patterns provide a wealth of input variables for the construction and improvement of fault diagnosis models. This enhances the predictive capability and accuracy of the models, thereby improving the efficiency of automated diagnostic systems and providing a basis for fault tracing.

**Figure 12:**
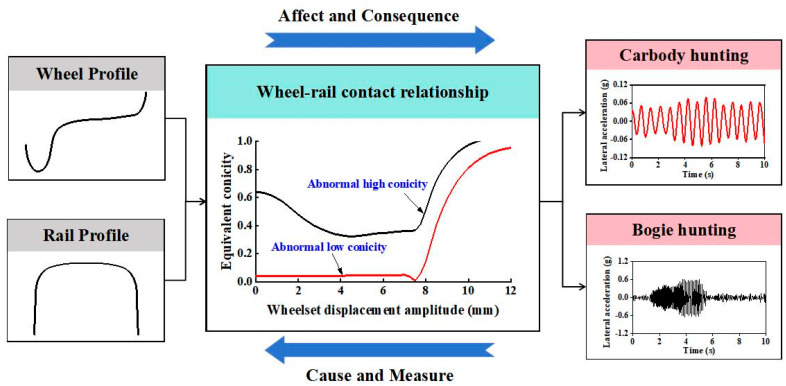
Hunting instability induced by abnormal wheel and rail wear.

The deterioration of the wheel-rail contact geometry is a major cause of hunting instability and significantly impacts the hunting stability of railway vehicles. The concept of equivalent conicity, derived as a linearized parameter of the wheel-rail contact system, has been proposed and widely adopted in the global railway industry to characterize and evaluate the wheel-rail contact condition. As shown in Figure 12, abnormal wear on the wheels and rails leads to deviations in the equivalent conicity, which in turn causes hunting instability. Therefore, the hunting stability of railway vehicles is closely related to the equivalent conicity of the wheelsets, which has been extensively researched by scholars. Wang [19] and Shi [4] observed hunting instability phenomena in bogies during long-term service tracking tests of high-speed trains. They pointed out that abnormal high conicity resulting from concave wear on the wheel tread is a key factor triggering instability. Chi et al. [73] conducted a simulation analysis to investigate the impact of excessive railhead grinding on wheel-rail contact matching and vehicle stability. They found that, under conditions of excessive railhead grinding, the contact points between the wheel and rail tend to cluster around the tread root and rail shoulder, leading to an abnormal increase in equivalent conicity. This abnormal conicity can trigger hunting instability in the bogie. Furthermore, deviations in wheel-rail matching conicity not only cause bogie hunting instability but, when excessively low, can also lead to high-amplitude oscillations and body hunting instability. Such issues typically arise early in the wheel tread wear process. Based on measured data and simulation analysis, Sun et al. [14,73,74] discovered that abnormal low conicity resulting from wheel tread flattening, excessive rail shoulder wear, and excessive rail cant are the causes of body hunting instability. Additionally, excessively high block hardness and sustained high-pressure operating conditions are identified as the causes of abnormal wheel tread wear. Feng et al. [75] investigated the body sway phenomenon following wheel re-profiling and found that this re-profiling method shifts the wheel profile towards the flange, leading to a reduction in equivalent conicity and consequently triggering body hunting motion. Additionally, variations in the friction coefficient within the wheel-rail interaction may also affect the stability of hunting motion. Li [76] conducted a multibody dynamics simulation study and found that when the wheel-rail friction coefficient is low, the vehicle is prone to head shaking.

Furthermore, for Wheel-rail high-speed vehicles, the suspension system typically employs a two-stage suspension mechanism, including primary and secondary suspensions. This design aims to ensure stable dynamic performance during high-speed operation while also enhancing overall operational safety and passenger comfort. Consequently, when suspension components experience functional failure or performance degradation, the critical speed of the vehicle can significantly decrease, leading to a higher likelihood of hunting instability. Jiang et al. [77] found that blockage of the damping valves significantly increases the damping force and dynamic stiffness of yaw dampers, which can potentially lead to severe low-frequency hunting motions and deteriorate the ride comfort of high-speed trains. This effect is particularly pronounced when the equivalent conicity of the wheel-rail system is low, especially if the damping valves in three or more locations of the yaw dampers are obstructed. In [78], Gao et al. discovered that cavitation phenomena in hydraulic yaw dampers (HYDs) cause them to generate damping force only during either the extension or compression stroke, leading to severe bogie hunting motions. Guo et al. [79] conducted experiments and analysis on a high-speed train exhibiting bogie hunting during the later stages of wheel wear. They identified that severe bogie hunting motions were primarily caused by abnormal stiffness and damping due to dynamic compression voids in the yaw dampers. At higher operating speeds, the vehicle system becomes highly sensitive to variations in component parameters. Wang et al. [80] examined the effects of slight variations in parameters, such as hydraulic fluid temperature, small installation clearances, and dynamic stiffness of the hydraulic fluid on the damping characteristics of yaw dampers through simulation analysis. These changes, in turn, affect the critical speed and ride comfort indices of the train. Xia [81] found that as the equivalent damping of the yaw dampers increased, the frequency of the bogie hunting motion continuously decreased. Furthermore, the frequency of the bogie hunting motion decreased with an increasing equivalent stiffness of the yaw dampers but eventually stabilized. Li et al. [7] conducted experiments and numerical analyses on the body sway of an intercity train. They indicated that body sway as a manifestation of body hunting instability and highlighted that low equivalent conicity, resulting from abnormal wheel tread wear, and reduced dynamic damping of the yaw dampers are primary causes of this instability. Moreover, the simultaneous occurrence of low equivalent conicity and diminished damping in the yaw dampers significantly reduces stability within the range of carbody sway speeds.

In addition to the effects of wheel-rail wear and the degradation of suspension components, vehicle structural damage, improper maintenance of the running track, and environmental changes are also major factors contributing to hunting instability. The nodal point of rotary arm refers to the connecting structure between train carriages that allows relative movement within a certain range, accommodating track curvature and irregularities. A nodal point of the rotary arm with more suitable stiffness can effectively reduce the vibration frequency and amplitude of the structure, thereby enhancing its vibration resistance. Song et al. [82] conducted a study on the impact of changes in longitudinal stiffness at the nodal point of the rotary arm on vehicle performance. The study concluded that increasing the longitudinal stiffness of the nodal point of the rotary arm can reduce the vehicle’s nonlinear critical speed and increase wheel-rail wear. However, it is important to note that the extent of its impact on vehicle performance is closely related to the wheel-rail matching conditions and the type of yaw dampers used. Track irregularities play a critical role in the vehicle operational safety, passenger comfort, and vibration response. When the carbody experiences swaying, the wheelset exhibits significant lateral displacement, increasing the lateral contact force between the wheel and the rail, thereby exacerbating wear on the rail gauge side. Under continuous external influences, the train tends to exhibit synchronous hunting motion at the same location. After a certain total weight has passed over the track, the rail gradually develops uneven alternating side wear. This side wear generates periodic excitations at the wheel-rail interface, further intensifying carbody hunting, thus creating a vicious cycle [83]. Zhang et al. [84] indicated that when there is periodic track irregularity in metro operating lines, especially when the track excitation frequency, bogie hunting frequency, and the natural swaying or rolling mode frequency of the carbody are simultaneously close, coupling between the bogie hunting motion and the inherent swaying or rolling motion of the carbody is likely to occur, intensifying the lateral oscillation of the vehicle. Li et al. [85,86] analyzed and found that the abnormal swaying of the carbody at operating speeds is a manifestation of frequency coupling resonance between the periodic track irregularity with a wavelength of 11–13 m, the bogie hunting frequency, and the carbody mode frequency. They demonstrated that maintaining a rail cant of about 1/40 and reducing the wear depth at the track gauge and gauge corner can improve the hunting stability of electric locomotives. Liu [87], through a combination of field and simulation tests, demonstrated that track geometry irregularities before intersections can induce hunting motion in trains. In addition to track irregularities, aerodynamic loads are another external excitation source inducing carbody swaying. Ding et al. [88] calculated and found that the trailing car, compared to the leading and middle cars, experiences a higher lift, lateral force, and yaw moment. They indicated that aerodynamic interference might be a key factor exacerbating the abnormal swaying of the trailing car in high-speed trains. In [6], Wang et al. demonstrated that the low-frequency hunting motion of the trailing car in high-speed trains can be induced by the lift-induced airflow generated during high-speed travel. They also identified the range of aerodynamic force coefficients that can trigger low-frequency hunting motion in high-speed trains. Sun et al. [89] indicated that vehicles are prone to secondary hunting in calm wind conditions, whereas primary hunting is more likely to occur under wind loading. They identified that a combination of higher equivalent aerodynamic lateral forces and greater aerodynamic lift creates the most unfavorable conditions for primary hunting. Furthermore, their study revealed that the duration of wind loading and track irregularities significantly affects the amplitude of limit cycle vibrations in vehicle hunting. Therefore, when assessing the operational safety of high-speed trains under strong wind conditions, it is essential to consider both the magnitude and duration of the actual aerodynamic forces and track irregularity excitations. In response to the lateral swaying of the trailing car that occurred when a certain type of 160 km/h urban EMU locomotive was running in a single-track tunnel, Hu et al. [90] pointed out that significant yawing moments in the aerodynamic forces and their coupling resonance with the vehicle’s hunting mode frequency are key contributors to the trailing car’s lateral swaying. They also suggested that optimizing the vehicle’s suspension parameters or increasing the damping ratio could help to alleviate the swaying issue in the trailing car.

The dynamic performance of railway vehicles during operation is influenced by a combination of various complex factors. Table 2 displays the influencing factors and their impact patterns as discussed in the research. These factors not only individually affect the vehicle’s operational status but may also interact collectively. For example, factors, such as wheel-rail equivalent conicity and damping force unloading of the anti-sway damper [7], as well as track conditions and wheel-rail profile compatibility [84], can lead to significant changes in vehicle dynamics and its evolution. Therefore, studying the influencing factors and their impact patterns on hunting instability is crucial for ensuring the safety and stability of vehicle operation. By thoroughly analyzing these factors, we can accurately identify the primary causes of hunting instability and implement targeted optimization measures to ensure that high-speed trains maintain smooth and safe operation in complex running environments. This not only enhances operational efficiency but also improves passenger comfort, contributing to the sustainable development of high-speed rail systems.

### 5.2. Dynamic Degradation Mechanisms of Hunting Instability

Since the advent of rail transportation, the issue of hunting motion has been a focal topic, resulting in a wealth of research achievements. However, most studies have concentrated on analyzing the critical speed of hunting instability and its influencing factors, with relatively fewer investigations into the evolution mechanisms of hunting instability. From the perspective of wheel-rail wear accumulation, hunting instability is a progressively evolving process characterized by dynamic coupling, transitioning from small-amplitude convergence to small-amplitude divergence, and eventually leading to large-amplitude instability or triggering alarms. This progression involves a gradual intensification of fault features from weak to strong. Moreover, the complex operating environment and variable working conditions have a significant impact on the abrupt changes and development of hunting stability. Therefore, it is very necessary to carry out an in-depth study on the behavioral system and evolutionary mechanism of hunting stability to provide theoretical guidance for precise fault assessments. Sun et al. [91] investigated the change rule of vehicle dynamic performance under a time-varying wheel-rail contact relationship. They found that an increase in equivalent conicity leads to a decrease in critical speed, which subsequently causes an increase in lateral wheel-rail forces and derailment coefficients, as well as higher lateral stability indices. In [92], Lin reached similar conclusions through their study on the impact of wheel concave wear on vehicle running performance. Polach et al. [93,94,95] utilized a nonlinear three-dimensional vehicle dynamic simulation model to study the evolution characteristics of railway vehicle hunting stability. They conducted a comparative analysis of existing stability evaluation methods and found that nonlinear wheel-rail contact geometric parameters significantly affect the evolution of the critical speed of the vehicle system and the limit cycle amplitude of the wheelset. Based on dynamic simulation modeling, Sun [60] quantitatively investigated the effects of bogie hunting and body hunting on vehicle dynamic performance. The study revealed that both bogie hunting and body hunting significantly degrade vehicle ride comfort and safety. Specifically, at 300 km/h, the lateral stability index of the vehicle body exceeds 3.0, and at 350 km/h, the derailment coefficient exceeds 0.8. In [79], Guo showed that bogie hunting instability degrades the vehicle’s lateral ride quality index. Experimental analysis on a roller test rig revealed abnormal vibrations around 9.5 Hz, leading to a higher lateral stability index for the vehicle. At a speed of 200 km/h, the lateral stability index exceeds 2.5, and at a speed of 350 km/h, the index reaches 3. Wen et al. [96] investigated the evolution characteristics of hunting stability in metro vehicles under service conditions. They pointed out that both wheel tread wear and a reduction in the wheel radius can exacerbate hunting instability and may lead to the formation of asymmetric hunting motion phenomena.

The interaction of multiple factors contributes to the onset of both bogie hunting and body hunting. As the service duration and operational speed increase, these phenomena markedly deteriorate the vehicle’s operational stability and safety, leading to a significant rise in lateral stability indices and derailment coefficients.

### 5.3. Common Features of Bogie and Carbody Hunting Instabilities

Constructing common features for characterizing hunting instability is essential for effectively monitoring and maintaining the dynamic performance of railway vehicles. This practice not only aids in identifying and taking precautions against both bogie and body hunting instability but also provides a solid technical foundation for their coordinated diagnosis. Such an approach enables timely and effective preventive and corrective measures during the early stages, thereby ensuring the safety and comfort of vehicle operations.

From the perspective of linear systems, hunting instability is a dynamic phenomenon caused by shifts in the system’s eigenvalues. Specifically, hunting instability arises when the eigenvalues transition from the negative real part plane (the stable region) to the positive real part plane (the unstable region). This transition results in significant alterations to the system’s modal parameters, thereby causing deviations in the vehicle’s operating state. Sun et al. [97,98] employed the modal continuous tracking method to investigate the modal features associated with hunting instability. Their study observed phenomena, such as frequency shifts, jumps in the damping ratio, and mode switching during carbody hunting. Based on the modal characteristics under hunting instability, Kritikakos et al. [99] achieved the early detection of carbody hunting. From the perspective of nonlinear dynamical systems, hunting instability is a typical bifurcation phenomenon. It manifests as the lateral motion of the wheelset diverging from a stable equilibrium point to a limit cycle of a certain amplitude when the vehicle’s speed crosses the critical threshold [100]. During this process, the vehicle’s vibration response exhibits several intrinsic characteristics, including harmonic signal autocorrelation [41], frequency capture cross-correlation [27,60], phase lag persistence [38], and periodicity in phase trajectories [43]. These characteristics are crucial for the accurate detection of hunting instability.

Failure mechanisms are fundamental to fault diagnosis, and the effectiveness of diagnostic methods ultimately depends on the thoroughness of the study of these mechanisms. As noted above, existing research has partially unveiled the mechanisms of hunting instability; however, a critical barrier remains between these mechanisms and the diagnostic processes. The key issues include the following: ① current mechanistic research has inadequately addressed the urgent need for diagnostic studies in the fine-tuned assessment and traceability of instability. Research oriented towards fault diagnosis remains insufficient and lacks a comprehensive descriptive system for characterizing hunting instability; ② simulation data derived from theoretical models often fail to accurately replicate the significant background noise encountered in practical engineering settings. High signal-to-noise ratio signals from these models present challenges for direct application in the development of diagnostic algorithms.

## 6. Challenges and Recommendations

### 6.1. Faced Challenges

Despite the promising results and excellent performance of the diagnostic methods for the early detection of hunting instability in high-speed trains as summarized in this paper, several challenges remain in practical applications. Key areas of discussion, along with potential solutions and future directions for development, are outlined below:

**(1) Limited Dataset Samples**

Although the condition monitoring system continuously gathers operational data, faults during the actual operation cycle of high-speed trains occur relatively infrequently. Furthermore, certain faults may not be detected in their early stages. Consequently, the majority of the collected data represents normal conditions, resulting in a limited amount of fault data. In fault diagnosis, small datasets can reduce diagnostic accuracy and model performance stability, complicate feature selection and engineering, and hinder the effective application of deep learning models, often leading to overfitting issues. Furthermore, small datasets can introduce bias and fairness issues, such as class imbalance and insufficient sample representativeness. Recently, many researchers have introduced deep learning-based data augmentation techniques, such as Autoencoders (AE) [101] and Generative Adversarial Networks (GAN) [102], to generate samples and expand datasets.

**(2) Imbalanced Datasets**

During their service life, high-speed trains predominantly operate in a stable state. Even when instability occurs, the types of instability are unlikely to encompass all possible states. Consequently, the input data used for diagnosing hunting instability are characterized by extreme class imbalance [34]. In fault diagnosis models, there is a tendency for the model to become biased towards classes with a larger number of samples during the fault identification process, potentially leading to overfitting in minority class samples. This results in the model’s inability to generalize effectively to real-world applications. Additionally, the data imbalance issue may hinder the accurate identification and evaluation of features that are useful for minority classes. This situation poses significant challenges for research on hunting instability detection using data-driven approaches, prompting extensive exploration by numerous scholars. Among these, the Denoising Diffusion Probabilistic Model (DDPM) [103] and its updated version, the reparameterized residual denoising diffusion probability model (ReF-DDPM) [104], have been demonstrated to be effective as novel data augmentation methods for addressing data imbalance issues in other fields.

**(3) Unlabeled Samples**

Large-scale and high-quality labeled data are prerequisites for the successful application of intelligent diagnostics [105]. Hunting instability exhibits complex dynamic characteristics, and the extensive data collected during the real-time monitoring of high-speed trains are challenging to label accurately in conjunction with data acquisition. As a result, the data often lack clear fault or anomaly labels. In the absence of labeled samples, training a supervised learning model demands considerable human effort and time for data annotation, which is highly costly. Furthermore, in specialized fields, such as hunting instability diagnosis, the quality of labeling directly affects model performance. Even with unsupervised learning methods, the effectiveness in handling complex pattern recognition and anomaly detection is limited, and these methods may struggle to accurately identify the diverse features of hunting instability.

In light of the aforementioned issues, some researchers [47] have proposed constructing vehicle dynamic models using Simpack to generate simulation data for various fault states for deep learning applications. Nevertheless, practical applications have exposed notable discrepancies between simulation data and actual data from high-speed trains. While simulation data, derived from precise mathematical modeling and controlled variables, provide theoretical predictions and parameter sensitivity analyses for vehicle performance under various operating conditions, simulated data are influenced by model assumptions and the accuracy of input parameters. It often involves simplifications compared to real-world high-speed trains and disregards external uncertainties, such as the track conditions, temperature, and wind. Consequently, it may not fully capture the complex actual operating environment and nonlinear effects, which can significantly impact the likelihood and manifestation of faults in real-world scenarios.

In summary, it is currently challenging to obtain a large amount of high-quality, labeled, multi-source fault data for high-speed trains under operational conditions, which can hardly support the construction of data-driven diagnostic models. Furthermore, simulation results derived from dynamics often do not directly guide the development of diagnostic algorithms.

### 6.2. Prospects for Migration-Assisted Hunting Instability Diagnosis

**Figure 13:**
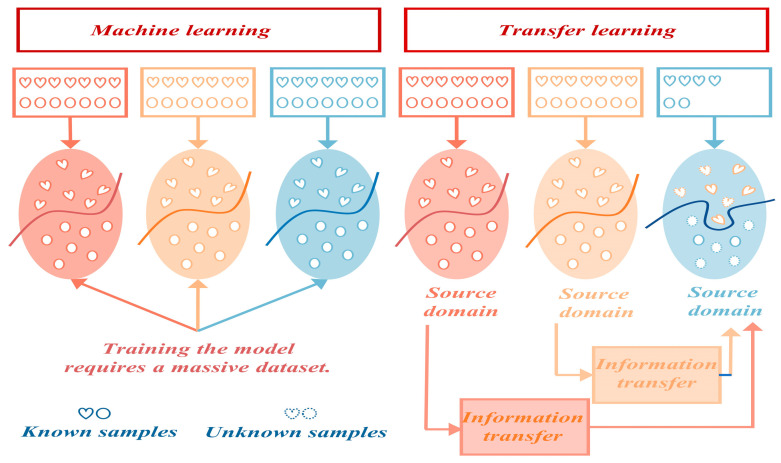
Machine learning vs. transfer learning.

To overcome these limitations, Transfer Learning (TL) presents a viable solution. TL aims to leverage data or knowledge from related domains (known as the source domain) to enhance learning in a new domain (referred to as the target domain). This method can effectively mitigate the problem of limited labeled fault data [106]. The distinction between TL and traditional machine learning approaches are depicted in Figure 13.

Transfer Learning (TL) has already demonstrated successful applications in various fields, including text classification, computer vision, biomedical research, and fault diagnosis for rotating and reciprocating machinery. Zhuang et al. [107] connected transfer learning with text classification issues by proposing a matrix-decomposition-based transfer learning framework to bridge the gap between different domains. This framework efficiently fits the data and addresses the challenge of dealing with similar but differently distributed data. To reduce the manual annotation of sensor data, Hu [108] proposed a transfer learning framework that automatically learns the correspondences between different sensor groups for identifying and predicting human activities. Also, to address the problem of insufficient labeled samples, Tang et al. [109] combined active learning and transfer learning for the classification of various medical data. Maqsood et al. [110] applied transfer learning to the medical imaging field for detecting different stages of Alzheimer’s disease. They fine-tuned an AlexNet model pre-trained on source domain data using instances from the target domain, achieving high accuracy. Facing the challenge of scarce data for phenotype-genome association prediction, Petegrosso et al. [111] proposed a transfer learning method based on a label propagation algorithm. Dong et al. [112] proposed a bearing fault detection method based on a convolutional neural network (CNN) and parameter transfer strategy for the small sample problem, which can significantly reduce the feature distribution difference and improve the fault identification performance. In the aforementioned studies, it has been demonstrated that transfer learning can effectively address the same issues encountered in the field of hunting instability fault diagnosis when applied to other domains. Additionally, transfer learning has shown greater effectiveness in classification and recognition tasks.

However, the application of transfer learning in the field of high-speed train hunting instability diagnosis is not yet widespread. Ning et al. [105] were the first to apply transfer learning to the diagnosis of hunting motion instability. They used the stable and unstable data obtained from simulation models as the source domain and the stable data from actual measurements as the target domain. By leveraging the high similarity between the two domains, they applied transfer learning to identify small-amplitude hunting in the bogie. Ning’s research provides a valuable reference and possibility for utilizing diverse types of simulated data to conduct transfer learning for hunting instability diagnosis. However, the study still has the following shortcomings: ① it still remains in the detection phase of hunting instability. ② It uses labeled data in the target domain, thus remaining within the realm of supervised learning. ③ It does not utilize measured instability data from the target domain, which may lead to discrepancies in data distribution and label space asymmetry, potentially affecting the performance of transfer-learning-based diagnostics.

**Figure 14:**
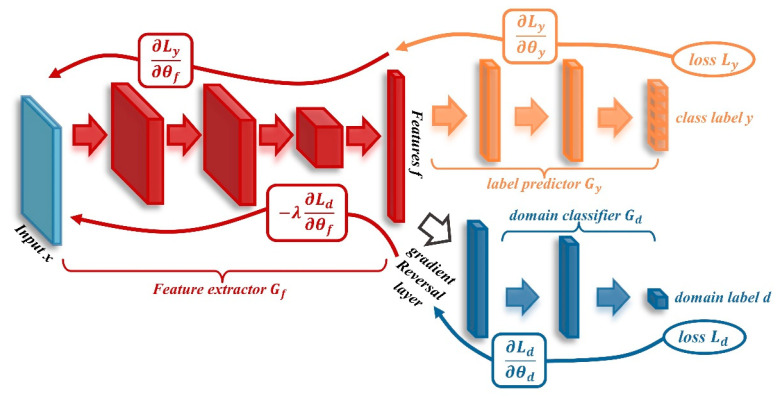
Domain adversarial neural network.

Adversarial learning methods based on deep domain adaptation have shown superior performance in the diagnosis of problems where the target domain is unlabeled and are widely applied in transfer diagnosis research. In 2015, Ganin and Lempitsky [113] proposed the Domain-Adversarial Neural Network (DANN), as illustrated in Figure 14. In this model, *G_f_* is the feature extractor, *G_d_* is the domain discriminator, and *G_y_* is the classifier. Specifically, *G_f_* and *G_d_* are connected through a gradient reversal layer.

DANN achieves alignment between the source domain and the target domain in the absence of labeled data in the target domain by utilizing a loss function, thereby enabling classification in the target domain. This approach provides a theoretical foundation and methodology for subsequent adversarial domain adaptation models. The loss function $L\left(\theta_{f},\theta_{y},\theta_{d}\right)$ is defined as follows:

(12) $$L\left(\theta_{f},\theta_{y},\theta_{d}\right)=L_{y}\left(\theta_{f},\theta_{y}\right)-\lambda L_{d}\left(\theta_{f},\theta_{d}\right)$$

(13) $$L_{y}\left(\theta_{f},\theta_{y}\right)=-\frac{1}{n_{s}}\sum_{i=1}^{n_{s}}\sum_{k=0}^{K_{s}-1}I\left[y_{i}^{s}=k\right]\log\left(G_{y}^{k}\left(G_{f}\left(x_{i}^{s}\right)\right)\right)$$

(14) $$L_{d}\left(\theta_{f},\theta_{d}\right)=-\frac{1}{n_{s}}\sum_{i=1}^{n_{s}}\log\left(G_{d}\left(G_{f}\left(x_{i}^{s}\right)\right)\right)-\frac{1}{n_{t}}\sum_{j=1}^{n_{t}}\log\left(1-G_{d}\left(G_{f}\left(x_{j}^{t}\right)\right)\right)$$

Xiao et al. [114] improved the loss function and weight assignment mechanism, achieving unsupervised bearing fault transfer diagnosis from the simulation domain to the experimental domain. To improve the loss function, DANN incorporates the Joint Maximum Mean Discrepancy (JMMD) into the original loss function. This modification addresses the challenge of aligning conditional distributions in the target domain, where labels are unavailable. The formula for JMMD is given as follows:

(15) $$\begin{array}{ll} \hat{D}\left(\theta_{f},\theta_{y}\right) & =\frac{1}{n_{s}^{2}}\sum_{i=1}^{n_{s}}\sum_{j=1}^{n_{s}}k\left({\hat{f}}_{i}^{s},{\hat{f}}_{j}^{s}\right)\cdot k\left({\hat{y}}_{i}^{s},{\hat{y}}_{j}^{s}\right)\\ & +\frac{1}{n_{t}^{2}}\sum_{i=1}^{n_{t}}\sum_{j=1}^{n_{t}}k\left({\hat{f}}_{i}^{t},{\hat{f}}_{j}^{t}\right)\cdot k\left({\hat{y}}_{i}^{t},{\hat{y}}_{j}^{t}\right)\\ & -\frac{1}{n_{s}n_{t}}\sum_{i=1}^{n_{s}}\sum_{j=1}^{n_{t}}k\left({\hat{f}}_{i}^{s},{\hat{f}}_{j}^{t}\right)\cdot k\left({\hat{y}}_{i}^{s},{\hat{y}}_{j}^{t}\right) \end{array}$$

The modified loss function incorporating JMMD is expressed as follows:

(16) $$L\left(\theta_{f},\theta_{y},\theta_{d}\right)=L_{y}\left(\theta_{f},\theta_{y}\right)-\lambda L_{d}\left(\theta_{f},\theta_{d}\right)+\mu \hat{D}\left(\theta_{f},\theta_{y}\right)$$

where λ and μ are regularization coefficients, and their growth trajectories are given by the following formulas:

(17) $$\lambda ,\mu \sim 2m/\left(1+e^{-Kt}\right)-m$$

where *m* represents the limit of the regularization coefficients, and *K* determines the rate of their growth.

A key prerequisite for effective transfer learning is minimizing the data distribution disparity and ensuring a symmetric label space between the source and target domains. The dynamics of high-speed trains are highly complex. Therefore, when applying transfer learning to the field of hunting instability fault diagnosis in high-speed trains, it is essential to perform detailed modeling of the train simulation model during the transfer process from the simulation domain to the experimental domain. This approach minimizes the discrepancies between simulation results and real-world conditions, ensuring the best possible transfer learning diagnostic performance.

In summary, the possibility of combining mechanistic models with transfer learning for fine-grained evaluation and fault tracing of hunting instability is proposed. This approach introduces new perspectives into the diagnostic research of hunting instability in high-speed trains, with the potential to establish a more adaptable and widely applicable diagnostic system for hunting instability.

## 7. Conclusions

Hunting instability not only affects the stability and comfort of train operations but can also lead to accelerated wear on tracks and train components, increasing maintenance costs and potentially causing safety incidents. As operating speeds increase, the risk of hunting instability also rises significantly, manifesting as more intense lateral vibrations and acceleration fluctuations. This paper reviews the mechanisms and diagnostic methods of hunting instability following the development and research process of fault diagnosis. It sequentially covers stability evaluation criteria, the early detection of small-amplitude hunting instability, refined assessment, fault tracing, and the mechanisms of hunting instability. The review synthesizes and analyzes existing research in this field, discussing the significance and limitations of each study. Finally, based on the current state of research, it explores remaining issues in depth and draws the following conclusions:

(1) Explaining the development process of China’s high-speed trains. As train operating speeds continue to rise and external factors evolve, there is an urgent need to address issues related to instability warnings, operational performance enhancement, and maintenance costs. These challenges highlight the pressing demand for improvements in both operational performance and the reform of maintenance systems for China’s EMUs.
(2) Summarize the criteria used by researchers for assessing the warning standards of hunting instability in high-speed trains, including both bogie hunting and body hunting instability. It provides a theoretical foundation for the early warning and prevention of train hunting instability, thereby ensuring the safety and stability of EMUs during high-speed operation. However, current evaluation standards typically rely on single indicators (such as lateral acceleration) and fixed thresholds to determine hunting instability. This approach does not adequately account for variations in the train speed, track conditions, load scenarios, or complex dynamics of high-speed trains, and it overlooks early-stage small-amplitude hunting. It is recommended to introduce multidimensional evaluation indicators and dynamically adjust the thresholds based on actual operating conditions to enhance the flexibility and adaptability of the standards.
(3) The existing research on the diagnosis of early-stage minor hunting instability in high-speed trains is organized from three perspectives: diagnostic signal sources, diagnostic features, and diagnostic targets. Although early-stage small-amplitude hunting instability involves small vibration amplitudes, the repeated micro-vibrations can exacerbate wear between the track and wheels and impose continuous fatigue stress on critical components. If not accurately identified and effectively controlled, this condition may gradually evolve into more severe hunting instability, further increasing the risk to train operations and potentially leading to more serious safety incidents. Therefore, it is crucial to develop and implement more sensitive and high-precision onboard monitoring systems to capture key data during train operations in real time. By integrating signal analysis with machine learning techniques, engineers and operators can promptly identify and address hunting instability issues at their early stages.
(4) To achieve the research goal of developing a comprehensive description system for hunting motion and precise fault identification, this study organizes and reviews the relevant research on the refined evaluation of bogie hunting instability and body hunting instability, as well as effective fault source tracing in these areas. Research in this area is currently limited and lacks depth. This is primarily because understanding the fault mechanism is fundamental to fault diagnosis, and the effectiveness of diagnostic methods ultimately hinges on the thoroughness of fault mechanism research. Existing studies mostly focus on diagnostics based on signal data, often neglecting the underlying mechanisms and characteristics of the fault itself, and consequently fail to establish a comprehensive fault assessment system and tracing mechanism. Therefore, it is essential to develop more sophisticated and high-precision simulation models that incorporate fault mechanisms. These models should predict the likelihood of hunting instability under varying speeds, loads, and environmental conditions, thereby providing data support for design improvements and operational strategies.
(5) Starting from the operational mechanisms of high-speed trains, it is crucial to conduct an in-depth study of the intrinsic evolution mechanisms of hunting instability. This involves organizing research from three perspectives: wheel-rail contact relationships, suspension systems, and external influencing factors. Such an approach is essential for developing effective preventive and intervention measures. In practical operations and development, to ensure optimal performance of trainsets and prevent hunting instability, engineers should focus on optimizing wheel-rail profiles and contact geometry, adjusting suspension system stiffness and damping parameters, and dynamically adjusting train speed and load distribution based on the real-time monitoring of track conditions and the train status. This approach helps to prevent triggering the critical conditions that lead to hunting instability.

## Figures and Tables

**Table 1:** Relevant standards for determining hunting stability.

Standard	Frequency Ranges	RMS Calculation Window	Threshold Value	Signal Source
UIC 518	$f_{0}\pm 2$ Hz	100 m in length with a step size not exceeding 10 m	$k(10+\frac{P_{0}}{3})/2$ kN	Wheel-rail guiding force/axlebox transverse force
			$(12-\frac{M_{b}}{5})/2$ $m\cdot s^{-2}$	Bogie frame acceleration
EN 14363	$f_{0}\pm 2$ Hz	100 m in length with a step size not exceeding 10 m	$k(10+\frac{P}{3})/2$ kN	Wheel-rail guiding force/axlebox transverse force
			$(12-\frac{M_{b}}{5})/2$ $m\cdot s^{-2}$	Bogie frame acceleration
49CFR 213	10 Hz	2 s	Not exceeding 0.3 g	Bogie frame acceleration
UIC 515-1	10 Hz	_	$\mathrm{Not}\;\mathrm{exceed}\;8\sim 10\;m\cdot s^{-2}$ continuously over six instances	Bogie frame acceleration
TSI RST HS 232	3~9 Hz	_	Not exceed 0.8 g continuously across 10 instances	Bogie frame acceleration
GB/T 5599-2019	0.5~10 Hz	_	$\mathrm{Not}\;\mathrm{exceed}\;8\;m\cdot s^{-2}$ continuously across six instances.	Bogie frame acceleration

**Table 2:** Influencing factors and patterns of hunting instability.

Reference	Influence Type	Influencing Factor	Influence Pattern	Affected Object
[4,19]	Wheel-Rail Contact Relationship	Wheel tread conical wear	Abnormal high conicity caused by wheel tread conical wear induces instability, and the instability increases with the conicity and tread wear.	Bogie hunting
[73]	Wheel-Rail Contact Relationship	Over-grinding of rail heads	Under over-grinding conditions, wheel–rail contact points concentrate on the tread root circle and rail shoulder, leading to abnormal increase in equivalent conicity.	Bogie hunting
[14]	Wheel-Rail Contact Relationship	Excessive rail shoulder wear and excessive rail cant	Excessive rail cant and over-grinding at the inner corner of the rail lead to extremely low wheel-rail contact conicity.	Carbody hunting
[74]	Wheel-Rail Contact Relationship	Wheel tread wear flatness	Flattened wheel tread causes abnormal low conicity in wheel-rail contact.	Carbody hunting
[75]	Wheel-Rail Contact Relationship	Wheel turning	The overall movement of the wheel profile towards the flange decreases the equivalent conicity.	Carbody hunting
[76]	Wheel-Rail Contact Relationship	Low wheel-rail friction coefficient	Low friction coefficient reduces adhesion between wheel and rail, leading to wheel hunting.	Carbody hunting
[77]	Suspension Component Damage	Blockage of yaw damper valve	Blockage of the damper valve significantly increases the damping force and dynamic stiffness of the yaw damper.	Carbody hunting
[78]	Suspension Component Damage	Cavitation in hydraulic yaw damper	Cavitation phenomenon leads to damping force generated only during a single stroke of extension or compression.	Bogie hunting
[79]	Suspension Component Damage	Compression air stroke in yaw damper	Compression air stroke in yaw damper causes abnormal stiffness and damping.	Bogie hunting
[80]	Suspension Component Damage	Parameter variations in yaw damper	Increased temperature lowers the dynamic viscosity of the oil, weakening the damping capability of the hydraulic yaw damper; larger attachment stiffness results in a greater reduction in effective damping coefficient; larger gaps cause greater inertial impacts.	—
[81]	Suspension Component Damage	Changes in equivalent damping and stiffness of yaw damper	Increased equivalent damping and stiffness decrease the frequency of bogie hunting motion.	Bogie hunting
[7]	Wheel-Rail Contact and Suspension Component Damage Interaction	Changes in equivalent conicity and dynamic damping	Reduction in yaw damper damping decreases the minimum damping ratio, with lower equivalent conicity leading to a larger reduction.	Carbody hunting
[82]	Vehicle Structural Damage	Change in nodal point of rotary arm stiffness	Increasing the longitudinal stiffness of the nodal point of the rotary arm can lower the vehicle’s nonlinear critical speed and increase wheel-rail wear.	Carbody hunting
[84]	Track Irregularity	Frequency resonance	Track excitation frequency, bogie hunting frequency, and carbody hunting or rolling modal frequency are simultaneously close.	Carbody hunting
[85,86]	Track Irregularity	Frequency resonance	Frequency coupling resonance occurs between track excitation frequency, bogie hunting frequency, and carbody modal frequency.	Carbody hunting
[87]	Track Irregularity	Track geometry misalignment	Track geometry misalignments before intersections can induce hunting motion in trains.	—
[88]	Aerodynamic Load	Aerodynamic vortex	The aerodynamic vortex at the rear of the train induces yaw frequency of the rear vehicle.	Carbody hunting
[6]	Aerodynamic Load	Lift flow	The lift flow generated by the HST rear car at high speed can excite its low-frequency hunting movement.	Carbody hunting
[89]	Aerodynamic Load	Aerodynamic force	Combined conditions of large equivalent aerodynamic lateral force and large aerodynamic lift are prone to primary hunting occurrence.	Carbody hunting
[90]	Aerodynamic Load	Aerodynamic force	Large yaw moment in aerodynamic force and its primary frequency coupled resonance with the whole vehicle hunting frequency.	Carbody hunting
